# A comparison between carbon footprint of water production facilities in the Canary Islands: groundwater resources vs. seawater desalination

**DOI:** 10.1007/s40899-022-00706-0

**Published:** 2022-07-17

**Authors:** Noelia Cruz-Pérez, Juan C. Santamarta, Isabel Gamallo-Paz, Jesica Rodríguez-Martín, Alejandro García-Gil

**Affiliations:** 1 Departamento de Ingeniería Agraria y del Medio Natural, Universidad de La Laguna (ULL), Tenerife, Spain; 2 Departamento Técnicas y Proyectos en Ingeniería y Arquitectura, Universidad de La Laguna (ULL), Tenerife, Spain; 3 Geological Survey of Spain (IGME-CSIC), C/ Ríos Rosas 23, 28003 Madrid, Spain

**Keywords:** Water production, Climate change, Canary Islands, Groundwater, Carbon footprint

## Abstract

The Canary Islands have a water culture tied to the exploitation of their groundwater by means of wells and water galleries. However, the growth of tourism, the increase in the local population and the development of agriculture have led to the emergence of new ways of obtaining water, such as the desalination of seawater. The presence of these desalination plants covers the entire archipelago except for the island of La Palma, and sometimes they function as a complement to water needs, while in other cases they are the only source of drinking water available. To study the environmental impact of the production of drinking water through the exploitation of the aquifer and the desalination of seawater, the carbon footprint methodology was used following the guidelines of the GHG Protocol. The result has shown that seawater installations have the largest carbon footprint, mainly due to the high electricity consumption in the islands and the electricity mix of the archipelago which, as it does not rely entirely on renewable energy sources, increases CO_2_ emissions into the atmosphere due to the production of drinking water in the islands.

## Introduction

Guaranteeing access to drinking water and energy production are two of the main challenges currently facing the world's population (Hickman et al. 2017). Both sectors, water and energy, require extensive economic and infrastructure deployment. In addition, supplying people with the drinking water needed for all human activities, as well as providing them with a stable energy service, nowadays require a sustainable management approach, to face the increasing uncertainties associated with climate change such as pandemics, natural disasters, reduced rainfall or increased temperatures (Nanduri and Saavedra-Antolínez 2013).

Climate change is an environmental process affecting the whole globe, mainly caused by anthropogenic greenhouse gases (GHG) emissions, which is responsible of the global warming effect observed in the last decades (Jakučionytė-Skodienė and Liobikienė 2021). GHG are gases that absorb and emit radiant energy within the thermal infrared range. Although GHG in their natural abundance are necessary to regulate the temperature of the planet, this system has been decompensated due to the emission of GHG of anthropogenic origin (Zhou et al. 2015). In this regard, the carbon footprint concept makes possible to assign an amount of GHG emissions to a given product, activity and/or service. By footprint concept provides a quantitative value of those GHG emissions allowing to make carbon footprint comparisons between different products, activities and/or services, thus facilitating the establishment of mitigation and improvement objectives of the products, activities or/and services studied.

Oceanic islands have been recognized for being places presenting a great attraction for tourism due to their weather conditions, as well as their impressive landscapes (Fonseca et al. 2014). In addition, agriculture is also considered as an important activity in oceanic islands due to their need for food sovereignty. Those factors, together with the urban settings, the water demand are especially high (Kourgialas et al. 2018). The hydric model of the islands varies according to their groundwater and surface water reserves, as well as their capacity to implement technologies such as water desalination, artificial recharge of aquifers or the use of reclaimed water for certain uses, which can alleviate the pressure on drinking water sources (Nijhawan et al. 2013).

In the Canary Islands, carbon footprint studies have been conducted mainly related to tourist infrastructures (Diaz Perez et al. 2018; Fernández-Latorre and Del Olmo 2011). Not as many studies are related to emissions related to the production of drinking water, which is the objective of this article which, in turn, is in line with the provisions of Sustainable Development Goal number 6, which guarantees Clean Water and Sanitation. Therefore, the main purpose of this work is to analyse the carbon footprint of these facilities, to investigate possible differences between groundwater facilities and desalination plants. This objective achievement would allow to understand the environmental impact caused by these water production facilities on the environment, identify which type of facility present the lower carbon footprint thus helping in the definition of management strategies and adopting decision making regarding water resource management plans in the Canary Islands and oceanic islands in general where water resources are scarce. This work investigated six desalination plants, four groundwater wells for water supply and two water galleries all distributed in the different islands of the Canary Islands. The carbon footprint has been calculated for the years 2019 and 2020.

### Study area: Canary Islands

**Fig. 1:**
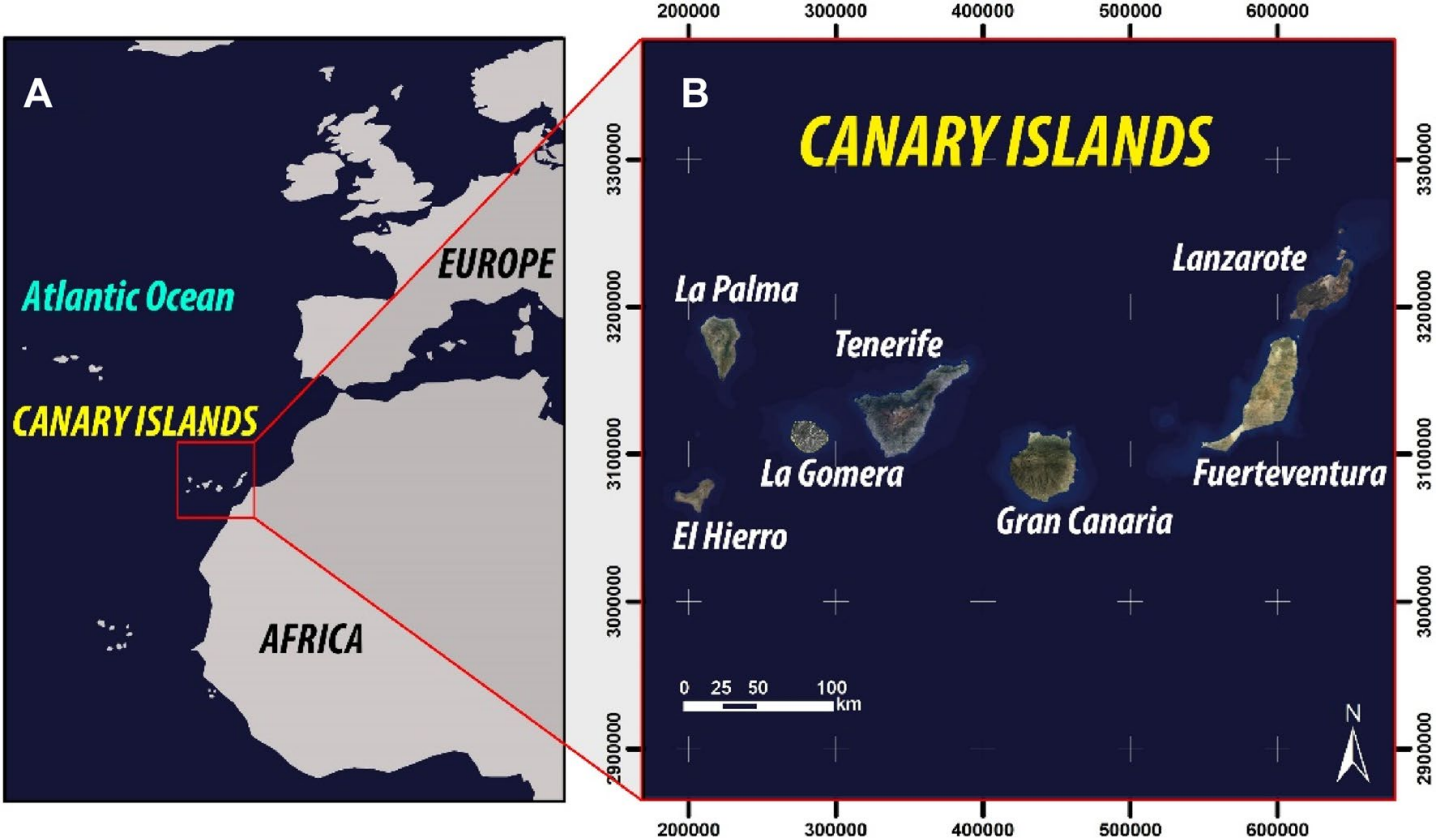
Geographical location of the Canary Islands. WGS 1989 Complex UTM Zone 28 N

The use of groundwater bodies in the Canary Islands is one of the factors that has facilitated the development of the main economic sectors of the islands, such as agriculture and tourism (Custodio et al. 2016a, b). The archipelago consists of a total seven islands (Fig. 1), each presenting its characteristic orography, geology, age, trade winds, proximity to the African continent, etc. All these aspects affect directly to the groundwater reserves and, therefore, conditioning the groundwater bodies’ response to the water demand of the islands in each island considered.

In the Canary Islands, groundwater is mainly captured through water galleries and/or wells, and there is an important culture and historical heritage related to these facilities in this archipelago (Santamarta et al. 2014). Since 1920 groundwater exploitation was widely developed in the western islands and *Gran Canaria* island, seeing their peak utilization between the 1950s and 1970s (Custodio et al. 2016a, b). Sustainable use of groundwater resources require withdrawals to be compensated by the natural recharge of the aquifer, i.e. by a percentage of the water that infiltrates the subsoil during precipitation episodes (Custodio et al. 2015). Water infiltration is largely affected by precipitation time distribution, and soil characteristics, becoming runoff in impermeable areas such as asphalt surfaces, and being able to infiltrate in green areas covered by vegetation (Neris et al. 2013). However, climate change is having a direct impact on the amount of precipitation expected in the future scenarios, which leads to consider artificial recharge of aquifers and desalinization.

In the Canary Islands, the existence of continental surface waters is limited to the existence of dams and reservoirs, whose purpose is to capture rainwater mainly for agricultural uses (Díaz et al. 2011). The islands of *Gran Canaria* and *La Gomera* are the territories where the greatest number of dams can be found in the archipelago, with a total of 69 and 39 dams, respectively (González Gonzálvez 2012).

The water model of the Canary Islands is different between the western and eastern islands. The eastern islands, influenced by their proximity to the African continent and their orography, have been pioneers in seawater desalination (García-Rodríguez et al. 2016). The first seawater desalination plants were built in *Lanzarote* and *Fuerteventura* islands in 1964 and 1974, respectively (Gómez-Gotor et al, 2018). Desalination plants have been slowly introduced in the rest of the archipelago becoming predominant on the island of *Gran Canaria*. Currently, all the islands in the archipelago present this type of water facilities, with the exception of La Palma Island. This situation has made necessary to include desalinated water plants as an important asset in the water cycle of the Canary Islands. However, water desalination technology requires large amounts of energy to produce drinking water, thus requiring a water-energy binomial approach to investigate the environmental impacts of desalination plants (Schallenberg-Rodríguez et al. 2014). Concern about the high energy consumption of reverse osmosis desalination plants in the Canary Islands has led to the implementation of energy recovery devices, with outstanding results in energy efficiency when using pressure exchanger (PX) (Arenas Urrea et al. 2019). Other options more related to renewable energy have also been studied in the Canary Islands, with the aim of finding out the feasibility of using solar and wind energy to achieve energy autonomy for small private desalination plants (Padrón et al. 2019), which are so common in the Canary Islands.

Accordingly, the production of energy from renewable resources offers an alternative to alleviate the pressure of this activity on the environment by reducing GHG emissions (Santamarta et al. 2022). In addition, desalination plants generate brine waste during its reverse osmosis operations that requires a correct management of its disposal (Kress et al. 2020). When it comes to renewable energy production in the Canary Islands archipelago, there are internationally recognized models of sustainability, as it is the case of *Gorona del Viento* in the *El Hierro* island (Frydrychowicz-Jastrzebska, 2018). The use of renewable energies becomes essential in islands rich in natural resources such as the sun, wind or geothermic (Rodríguez et al. 2021).

## Methodology

**Fig. 2:**
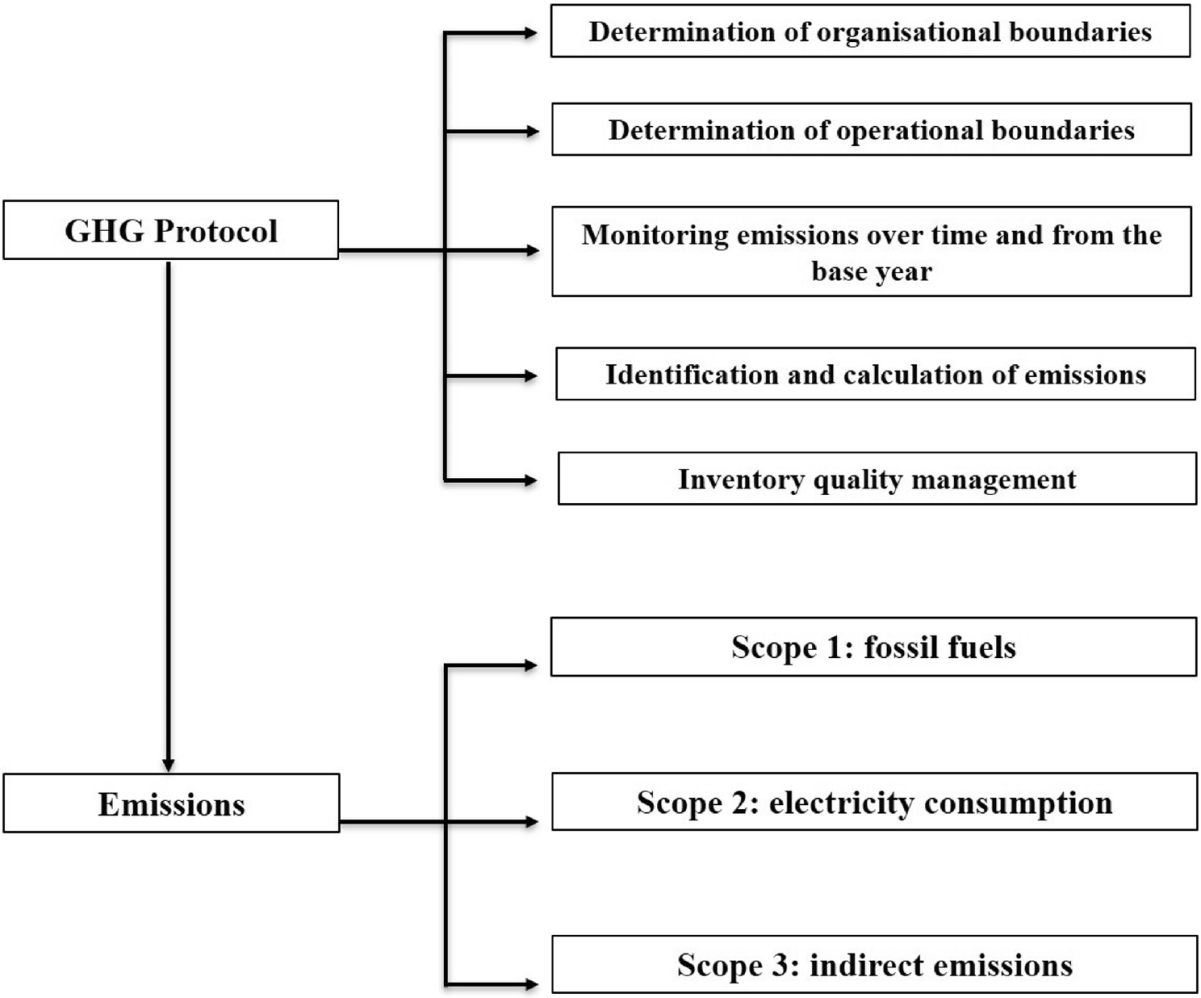
Methodology flowchart of the GHG Protocol that has been followed

**Fig. 3:**
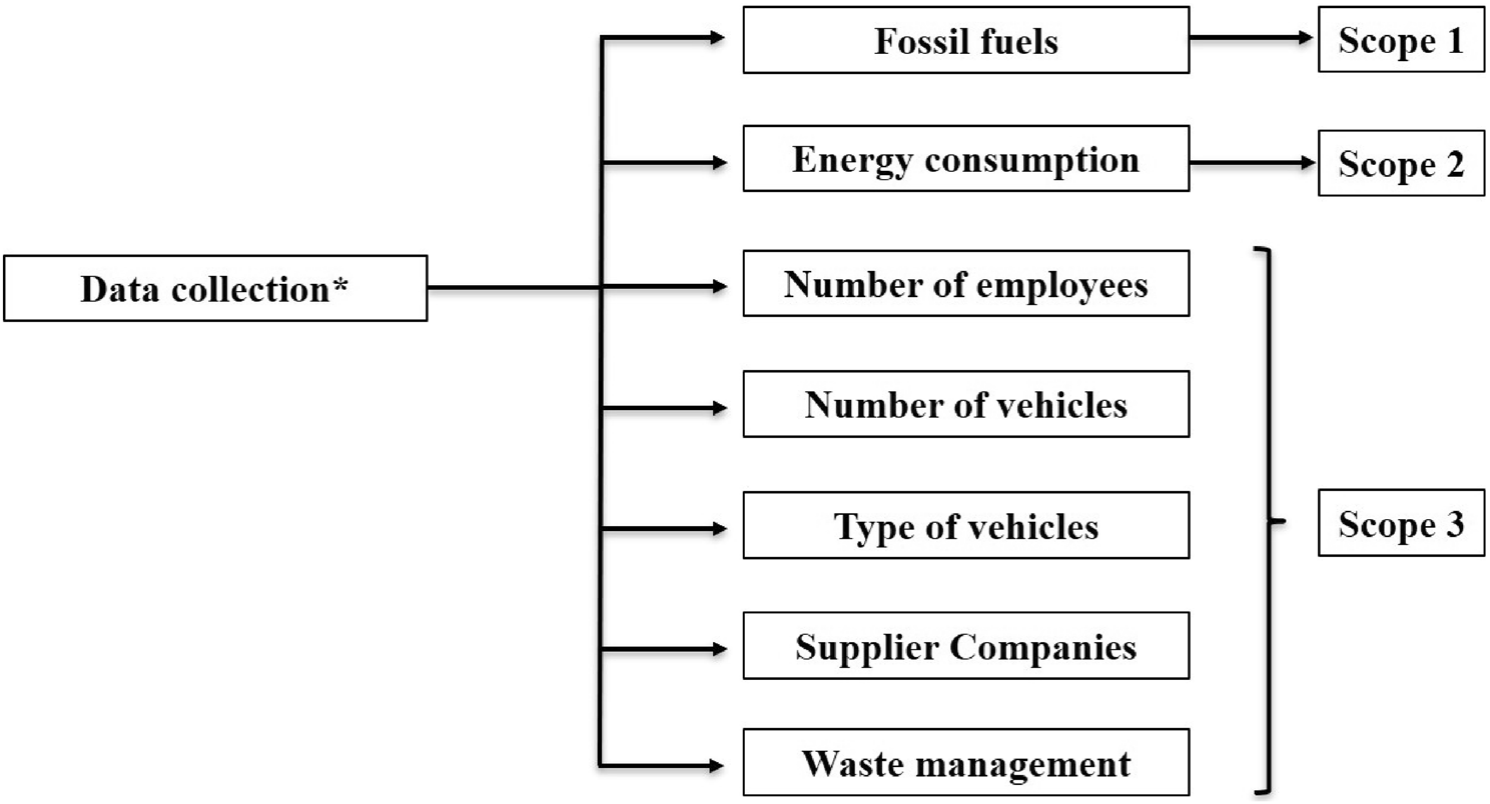
Relationship between the groups of data collected and the scopes of the GHG Protocol. *Data collected from the surveys of the studied companies under request

The carbon footprint makes it possible to identify the sources of GHG emissions in the manufacture of a product, the provision of a service and/or the development of an activity or event (Banhardt and Hartenstein 2019). To differentiate between the sources, the GHG Protocol (Bhatia et al. 2011; Fong et al. 2014) was used as a widely accepted standard to assess direct GHG emissions related to the use of fossil fuels directly by the company under study (scope 1), emissions related to the company's electricity consumption (scope 2) and indirect emissions corresponding to fossil fuels or other sources to be considered (scope 3) (Fig. 2). In order to obtain the most representative data, a survey was conducted to the installation management of the studied desalination plants, wells and water galleries where they provided the information needed to calculate the carbon footprint for each scope (Fig. 3).

Once the sources were defined, the emissions were counted and transformed into tons of carbon dioxide equivalent, using emission factors published by national official agencies. The data collected by the facilities studied are consumptions, with different units of measurement, generally in litres in the case of fossil fuels, or they can be km travelled and type of vehicle in the case of cars, and in the case of electricity the unit is kWh. Therefore, it is necessary to use emission factors proposed by national entities, in order to convert this consumption data into emissions data in the unit of measurement of the carbon footprint, which is the ton of carbon dioxide equivalent (tCO_2_eq). Therefore, when annual consumption data are obtained, they are divided by type of scope and converted into tCO_2_eq using the official factors, thus being able to study emissions and establish comparisons between facilities.

The GHG Protocol methodology was used to calculate the carbon footprint and subsequent comparison of the groundwater collection and seawater desalination facilities (Hickmann 2017). The GHG Protocol allowed to avoid heterogeneities in the methods and principles used for the calculation of GHG emissions of internationally accepted companies and organisations. The methodology of this system is based on the following points: (1) Determination of organisational boundaries; (2) Determination of operational boundaries; (3) Monitoring of emissions over time and from the base year studied; (4) Identification and calculation of emissions; and (5) Inventory quality management.

The objective of implementing the GHG Protocol methodology to determine greenhouse gas emissions to the atmosphere, in the annual operations of a company, is useful to identify the main sources of greenhouse gas emissions, and thus be able to implement mitigation measures. In other words, by calculating the carbon footprint it is possible to determine, by type of scope, where the main emissions are being produced and whether these are due to the company's own consumption of fossil fuels, whether in vehicles or in fixed installations (scope 1) or whether they refer to indirect emissions linked to electricity consumption (scope 2). It is in electricity consumption where a company's emissions tend to skyrocket, as long as the company's electricity mix does not include 100% renewable energies, or the percentage of these is very low. It is therefore vital to focus on energy production, which is closely linked to the production and treatment of drinking water (Santamarta et al. 2022).

The facilities studied in the islands were as follows: three desalination plants in *El Hierro*, one desalination plant in *Gran Canaria*, one in *Fuerteventura* and one in *Tenerife*. With regard to groundwater, two wells were studied in *El Hierro*, one well in *Gran Canaria*, one well in *Tenerife*, a water gallery in *Tenerife* and another water gallery in *Gran Canaria*. The carbon footprint has been calculated for the years 2019 and 2020, these years would also allow to preliminary identify COVID-19 pandemic effects on the production of drinking water in the archipelago.

## Results and discussion

**Fig. 4:**
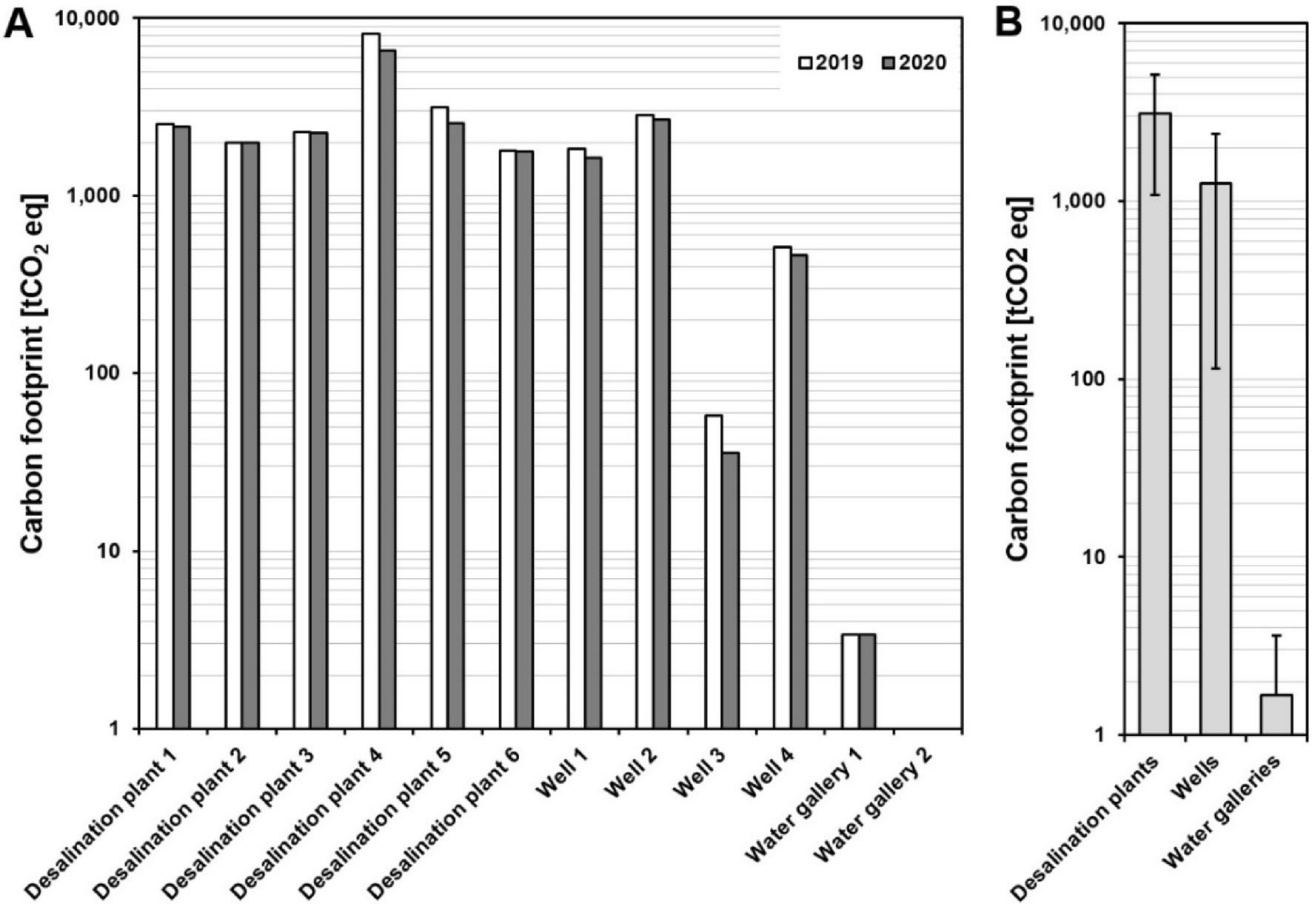
**A** Difference between the carbon footprints of water production installations in 2019 and 2020. **B** Averaged carbon footprint of each type of water production facility, whiskers represent the standard deviation

Results obtained after studying the carbon footprint of six desalination plants, four wells and two water galleries in the Canary Islands, for the years 2019 and 2020, are presented in Table 1. In addition, the difference in the footprints for these two years can be seen in Fig. 4.

**Table 1:** Calculated carbon footprint for desalination plants and groundwater production facilities (years 2019 and 2020)

	2019				2020			
	Scope 1	Scope 2	Scope 3	Carbon footprint	Scope 1	Scope 2	Scope 3	Carbon footprint
Units	[tCO_2_ eq]	[tCO_2_ eq]	[tCO_2_ eq]	[tCO_2_ eq]	[tCO_2_ eq]	[tCO_2_ eq]	[tCO_2_ eq]	[tCO_2_ eq]
Desalination plant 1	0.4	842.3	1680.1	2522.9	0.4	762.6	1680.1	2443.1
Desalination plant 2	0.6	317.9	1680.1	1998.6	0.6	304.1	1680.1	1984.9
Desalination plant 3	1.4	602.6	1680.1	2284.2	1.4	584.2	1680.1	2265.7
Desalination plant 4	0.0	6171.0	1982.9	8153.9	0.0	4580.9	1982.9	6563.8
Desalination plant 5	0.4	1483.5	1680.5	3164.3	0.4	894.7	1680.5	2575.6
Desalination plant 6	4.4	1436.5	350.1	1791.1	4.6	1416.9	350.1	1771.7
Well 1	0.0	317.0	1512.2	1829.2	0.0	115.6	1512.2	1627.8
Well 2	0.0	495.0	2352.2	2847.2	0.0	315.1	2352.2	2667.3
Well 3	0.0	58.1	0.0	58.1	0.0	35.8	0.0	35.8
Well 4	0.0	174.2	336.0	510.3	0.0	124.7	336.0	460.8
Water gallery 1	0.0	0.0	3.4	3.4	0.0	0.0	3.4	3.4
Water gallery 2	0.0	0.0	0.0	0.0	0.0	0.0	0.0	0.0

To obtain the data, a survey was provided to the managers of the facilities studied, requesting the following information: (1) scope 1, consumption of fossil fuels in vehicles and in fixed installations (note that companies do not always have their own vehicles destined for the installation); (2) scope 2, electricity consumption of the facilities, as well as the company supplying the energy, since the electricity mix is the conversion value with which we obtain the emissions linked to electricity consumption (if the energy production of the supplying company is sometimes 100% renewable energy, the electricity mix would be zero, and there would be no emissions linked to electricity consumption, which would be very positive since scope 2 is generally the highest of all); (3) scope 3, the consumption of fossil fuels by the vehicles of agents indirectly linked to the facility under study (i.e. workers, suppliers and waste management in this case) is of particular interest.

In general terms, and with respect to each of the scopes that make up the carbon footprint, the following can be observed:

- *Scope 1* The consumption of fossil fuels by the concession companies studied is minimal or even non-existent. They are restricted to generators or similar devices, and in all cases their consumption is insignificant or non-existent. With regard to company vehicles, there are, in general, vehicles associated with one or more installations, mainly for maintenance and overhaul work. Also, a study conducted in a desalination plant in California concluded that there was no CO_2_ production from the facilities operation used in water transportation (Han et al. 2021).
- *Scope 2* Electricity consumption is the most important activity in the generation of emissions, due to the importance of the annual electricity consumption of installations such as wells and desalination plants. In fact, the major costs for energy consumption are located in the high-pressure pumps of the desalination plants (Leon et al. 2021).
- *Scope 3* It has been considered the routes of vehicles that are not owned by the company under study, but which make trips related to the company. Company employees, companies supplying products and/or services and waste management have been included. Since each vehicle type is associated with a certain amount of emissions per km travelled, the emissions of these vehicles and their journeys have been obtained on an annual basis for each facility. Regarding to waste management, desalination usually yields two products, fresh water and brine (water with high salinity and reject concentrate (Mavukkandy et al. 2019). This brine needs to be treated before sending it back to the ocean as a very diluted salt water. It has been observed that this scope, together with scope 2, is one of the most important in terms of emissions, as (especially the larger facilities) have a large number of workers and supplier companies. However, it should be considered that the habits of the workers as well as their type of vehicle may change throughout the year studied, which could lead to a possible error in the results obtained in this scope.

In the case of water galleries, these are characterised by their very low carbon footprint, as they are constructed in such a way that the water can be extracted by gravity from the aquifer. In this way, there is no energy consumption associated with their exploitation, with scope 1 and 2 being very low or non-existent. As for the wells, it is observed that they are linked to pumping facilities, since they are vertical works that seek groundwater, it is inevitably necessary to pump to bring the water to the surface. Depending on the depth, topography, volume or technology, the energy consumed will be different (Wakeel et al. 2016). Therefore, wells do have emissions associated with scopes 1 and 2, however, they tend to have a much smaller footprint (Fig. 4B) than seawater desalination plants (compared to other drinking water production facilities).

**Fig. 5:**
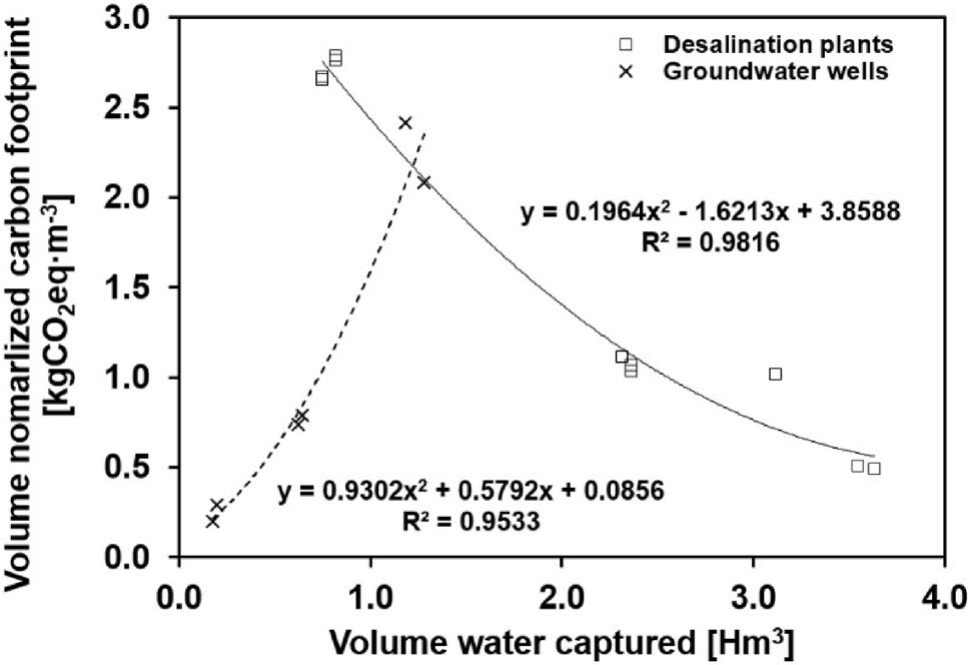
Volume normalized carbon footprint as a function of volume water captured by the different water production facilities. Well 1 and desalinization plant 4 were removed from the graph

The normalized carbon footprint by volume of water captured is shown in Table 2. Normalized carbon footprints from desalination 4 and well 1 are outliers from the tendency observed in the rest of the facilities. Desalination plant 4 shows one of the best normalized carbon footprints of 0.5 kgCO_2_eq·m^−3^ by far capturing more volume of water than the rest of the investigated facilities together. On the other hand, well 4 present the highest carbon footprint with 77.5 kgCO_2_eq·m^−3^, in this case with the lowest captured volume in all investigated facilities. From this observation it would be deduced that the higher is the volume of water processed, the lower is the carbon footprint per volume of water processed. However, the change of normalized carbon footprint values with the extracted volumes (Fig. 5) for the rest of facilities show different tendencies for the different production systems investigated. From this figure, it can be concluded that for less than 1.2 Hm^3^ groundwater wells present lower carbon footprint per unit of extracted volume increasing as the extracted volume increases. On the contrary, for facilities processing more than 1.2 Hm^3^ the normalized footprint tends to decrease as the processed volume of water increases when desalination facilities are considered.

**Table 2:** Calculated carbon footprint for desalination plants and groundwater production facilities (years 2019 and 2020)

	Volume captured		Carbon footprint		Volume normalized carbon footprint			
Units	Hm^3^		tCO_2_eq		tCO_2_eq Hm^−3^		kgCO_2_eq m^−3^	
Year	2019	2020	2019	2020	2019	2020	2019	2020
Desalination plant 1	2.364	2.364	2522.9	2443.1	1067.2	1033.5	1.1	1.0
Desalination plant 2	0.748	0.748	1998.6	1984.9	2672.0	2653.6	2.7	2.7
Desalination plant 3	0.820	0.820	2284.2	2265.7	2785.6	2763.1	2.8	2.8
Desalination plant 4	12.767	13.580	8153.9	6563.8	638.7	483.3	0.6	0.5
Desalination plant 5	3.117	2.314	3164.3	2575.6	1015.2	1113.0	1.0	1.1
Desalination plant 6	3.548	3.631	1791.1	1771.7	504.8	487.9	0.5	0.5
Well 1	0.084	0.021	1829.2	1627.8	21,776.3	77,516.2	21.8	77.5
Well 2	1.180	1.280	2847.2	2667.3	2412.8	2083.8	2.4	2.1
Well 3	0.198	0.177	58.1	35.8	293.3	202.2	0.3	0.2
Well 4	0.645	0.623	510.3	460.8	791.1	739.6	0.8	0.7

Seawater desalination plants have the largest carbon footprint of all those studied in the integral water cycle in the Canary Islands. It is undeniable that seawater desalination plants have made life easier for people in the Canary Islands, enabling local development and the expansion of tourism in all the islands (Sadhwani and Veza 2008). However, this high carbon footprint is largely linked to scope 2, i.e., electricity consumption. Therefore, it is recommended to the stakeholders contracting electricity supply with electricity mix is equal to zero or lower as possible.

There are facilities where there is a strong water-energy binomial, such as desalination plants and, to a lesser extent, wells. This approach has made it possible to see the carbon footprint reduction in 2020. This decrease in the carbon footprint could be explained since those facilities respond more to tourist demand than residential populations, together with significantly varied flows treated due to the pandemic (both to generate drinking water and to process the treated flows). Similarly, the reduction in the carbon footprint is not only due to the effect of the pandemic on the production and treatment of water, but also to improvements in the electricity mix of the energy supply companies, as well as the environmental measures being introduced by the companies that manage water in the Canary Islands.

In the Canary Islands, only 7% of the energy produced in the archipelago comes from renewable sources (Gils and Simon 2017). It is therefore necessary to increase this percentage, as well as to reduce electricity consumption and, of course, dependence on external sources. It should be highlighted that in the Canary Islands, 98% of the oil needed to produce energy by burning traditional fuels comes from overseas (Schallenberg-Rodríguez et al. 2014).

Every day 600,000 m^3^ of water are desalinated in the Canary Islands. Thanks to previous studies on the islands, it is known that the energy consumption of the desalination plants is approximately 4.80 kWh/m^3^. Therefore, we are referring to approximately 3000 GWh consumed annually in the archipelago for the production of desalinated water. Seawater desalination plants see their carbon footprint increase mainly due to scope 2, which accounts for emissions from electricity production. Therefore, until electricity suppliers fully incorporate renewable energies in their energy production, seawater desalination will continue to be linked to high GHG emissions (Diaz Perez et al. 2018).

Regarding the economic aspects of each of the water supplies considered in this study, the costs of installing and extracting water from a desalination plant are the highest considering the maintenance of the infrastructure compared to the wells and water galleries. For well construction, it is necessary to perforate and in volcanic terrains that could mean about 300–400€ per lineal meter (Santamarta 2016), but it requires less maintenance and once is constructed the costs are rapidly recovered. According to the national hydrologic plans for the islands, the mean cost of the groundwater per home is approximately 0.4€/m^3^. In case of desalination water, it was estimated in 0.50–0.60€/m^3^ (García Latorre et al. 2015).

## Conclusions

In the Canary Islands a strong water-energy nexus exists, especially on those islands that rely entirely on seawater desalination for drinking water. In terms of carbon footprint, scope 1 has been very low or non-existent in almost all cases, which would be totally eliminated if fossil fuels were no longer used. On the other hand, the high scope 2 obtained in seawater desalination plants could be reduced or eliminated if renewable energy sources were implemented in the desalination plants themselves, or if electricity supply were contracted to come entirely from renewable energy sources, since this supply would have an emission factor equal to zero, thus eliminating the emissions associated with scope 2.

One of the other conclusions drawn from the study of the carbon footprint of desalination plants and groundwater collection facilities in the Canary Islands is that the carbon footprint of desalination plants is higher than that of wells and water galleries. Also, Water galleries are positioned as the most energy efficient installations, but it must be borne in mind that they drain the aquifer, and therefore measures must be taken to guarantee its recharge. Consequently, it is considered that measures related to the integral water cycle in the archipelago should be taken, which would reduce emissions from all installations.

The future of the Canary Islands in terms of ecological transition involves increasing the use of renewable energies, especially in the water sector, which is positioned as one of the largest energy consumers in the Islands.

## Data Availability

The data that support the findings of this study are available from the corresponding author, upon reasonable request.
